# MiR-181a-5p Delivered by Adipose-Derived Mesenchymal Stem Cell Exosomes Alleviates *Klebsiella* pneumonia Infection-Induced Lung Injury by Targeting STAT3 Signaling

**DOI:** 10.1155/2022/5188895

**Published:** 2022-12-14

**Authors:** Ren-Jing Hu, Xiao-Chun Chen, Lei Xu, Xiao-Hong Rui, Lin Wan, Jie Lu, Jun Liu, Hao Pei

**Affiliations:** ^1^Department of Laboratory Medicine, Wuxi Second People's Hospital Affiliated to Nanjing Medical University, Wuxi City, 214000 Jiangsu Province, China; ^2^Department of Laboratory Medicine, Taizhou Second People's Hospital, Taizhou City, 225411 Jiangsu Province, China; ^3^Department of Oral and Maxillofacial Surgery, Wuxi Stomatological Hospital, Wuxi City, 214001 Jiangsu Province, China; ^4^Department of Laboratory Medicine, Wuxi Fifth People's Hospital, Wuxi City, 214000 Jiangsu Province, China

## Abstract

**Background:**

*Klebsiella pneumoniae* (*K. pneu*) is a leading cause of gram-negative pneumonia, which requires effective treatment. Adipose-derived mesenchymal stem cell- (ADSC-) derived exosomal microRNAs (miRNAs) have presented the inhibitory effect of multiple diseases. However, the function of ADSC-derived exosomal miRNAs in *K. pneu* remains unclear.

**Aim:**

In this study, we aimed to explore the effect of ADSC-derived exosomal miR-181-5p on *K. pneu* infection-induced lung injury.

**Methods:**

C57BL/6 mouse model was established by infection of *K. pneu*. ADSCs and exosomes were extracted and characterized in vitro. The translocation of ADSC-derived exosomes to bone marrow-derived macrophages (BMDMs) was detected. The level of miR-181a-5p was detected by real-time PCR. The secretion of inflammatory factors was determined by ELISA. The interaction between miR-181a-5p with STAT3 was identified.

**Results:**

We successfully isolated the ADSCs that express positive markers CD90 and CD105 rather than CD31 and CD45. The exosomal miR-181a-5p secreted by ADSCs were internalized by BMDM and *K. pneu* infection stimulated the miR-181a-5p level in bronchoalveolar lavage fluid (BALF) and BMDM. ADSC-derived exosomal miR-181a-5p repressed pulmonary outgrowth and dissemination of *K. pneu* infection in mice, repressed cellular infiltration in lung tissue, and attenuated the inflammasome activity and the levels of IL-1*β* and IL-18 in the lung. Mechanically, miR-181a-5p was able to inhibit STAT3 expression at posttranscriptional levels and repressed Nlrp3 and Asc expression in BMDM.

**Conclusion:**

Consequently, we concluded that ADSC-derived exosomal miR-181a-5p alleviated *Klebsiella* pneumonia infection-induced lung injury by targeting STAT3 signaling. ADSC-derived exosomal miR-181a-5p may serve as a potential candidate for the treatment of *Klebsiella* pneumonia infection-induced lung injury.

## 1. Introduction

*Klebsiella pneumoniae* (*K.pneu*) is a gram-negative encapsulated bacterium that causes a majority of hospital acquired infections, among which over 10 percent are pneumonia, and is steadily increasing as a common case for community-acquired infection of the respiratory tract [1]. *K.pneu* is both an opportunistic pathogen and a commensal bacterium in the gastrointestinal tract and can disseminate to secondary locations, hence is able to cause a wide range of infections, including bacteremia, pneumonia, sepsis, and urinary tract infection [2–5]. *K.pneu*-caused infections commonly occurred with remarkable accumulation of immune cells such as macrophages and neutrophils, as well as high levels of proinflammatory cytokines (termed as “cytokine storm”) due to dysregulated immune response, which leads to acute lung injury [6–8]. Noteworthy, *K.pneu* is emerging as a mortal threat for human, since most clinical strains are resistant to multiple antibiotics and some even show resistance to all available antibiotics [9, 10]. Hence, the need for new therapeutics to cope with *K.pneu* infection is urgent.

Mesenchymal stem cells (MSCs) are gradually developing into a promising therapeutic tool for diseases due to multiple advantages, including the relatively simple isolation procedure, low immunogenicity, self-renewal ability, and multipotency [11–13]. Moreover, MSCs are capable of secreting various angiogenic, anti-inflammatory, and antibacterial mediators that participate in immune response [11]. Mesenchymal stem cell (MSC) is also recently regarded as a potential new approach of immune modulatory therapy for pneumonia [14, 15]. Besides, adipose-derived mesenchymal stem cells (ADSCs) also have shown reliable and promising clinical application potential [16, 17]. It is suggested that ADSCs are able to differentiate into various cell types and secrete various cytokines that participate in the regulation of angiogenesis, wound healing, immunoregulation, and tissue regeneration [17]. In addition to cytokines, ADSCs also release exosomes, a form of extracellular vesicles that is capable of delivering mRNA, lipid, proteins, and microRNAs (miRNAs), which hence facilitates cell communication and participates in various functions of cells [18–20].

MiRNAs are a type of endogenous short noncoding RNAs with a length of 20 to 24 nucleotides, function through targeting the UTR region of mRNAs, and have been extensively studied in inflammation including *Klebsiella* pneumonia [21–23]. The miR-181 family has been indicated to be involved in the development of multiple pathological processes including cancer and neurodegeneration [24]. Qu et al. suggested that exosomes derived from miR-181-5p-modified ADSCs attenuated fibrosis, through activating autophagy [25]. Moreover, miR-181-5p was also recently disclosed to mediate IL22 regulated immune response by targeting STAT3 [26]. It has been reported that inhibiting STAT3 expression represses *K.pneu* [27]. However, the regulatory role of miR-181-5p in *Klebsiella* pneumonia is not clarified. In this study, we aim to exploit the function of ADSC-derived exosomal miR-181-5p in *Klebsiella* pneumonia and may provide a potential therapeutic approach for the treatment of *Klebsiella* pneumonia.

## 2. Methods

### 2.1. Bacteria and Materials

Lipopolysaccharide (LPS), PKH26 Fluorescent Cell Linker Kits (Sigma-Aldrich), and exosomal inhibitor GW4869 were obtained from Sigma (USA). The miR-181-5p mimics, inhibitors, Cyc3-labeled pre-miRNA, and negative control cel-miR-67 were designed and synthesized by GenePharma (China). The transfection reagent Lipofectamine 2000, cell lysis buffer RIPA, and TRIzol were obtained from Invitrogen (USA). The primary antibodies against IgG1, CD31, CD45, CD90, CD105, CD63, CD9, STAT3, CD68, and *β*-actin were ordered from Abcam (USA).

The *Klebsiella pneumoniae* (*K. pneu*) was purchased from the American Type Culture Collection (ATCC) and were grown overnight in a Luria-Bertani medium (Sigma) in rotation at 200 rpm at 37°C incubator. The bacteria were then resuspended in PBS and serially diluted. The concentration of *K. pneu* was measured at OD 600 nm and was diluted to appropriate colony forming unit (CFU) concentrations for subsequent experiments.

### 2.2. Isolation and Identification of Adipose-Derived Mesenchymal Stem Cell (ADSC)

Adipose tissue-derived mesenchymal stem cells (ADSCs) were isolated from the adipose tissue of healthy mice and cultured in Dulbecco's Modified Eagle's (DMEM, Gibco, USA) medium added with 10% fetal bovine serum (FBS, Gibco, USA), 1% penicillin/streptomycin (SolarBio, China), and 2 mM L-glutamine (Sigma). The ADSCs were used within three passages. The cells were stored in liquid nitrogen and thawed to obtain cryopreserved cells.

The ADSCs were labelled by antibodies against IgG1, CD31, CD45, CD90, and CD105 and detected by FACS in a flow cytometer (BD Biosciences). The differentiation of ADSCs to osteoblast or adipocytes was determined by Alizarin Red S and Oil Red O staining (Beyotime, China) following the manufacturer's protocols, respectively.

### 2.3. Isolation of Bone Marrow-Derived Macrophages (BMDMs)

The BMDMs were isolated following a classic protocol previously published [28]. Briefly, the femur/tibia of the mice were harvested and flushed with PBS to collect bone marrow cells. After RBC lysis, cells were centrifuged, washed, and set up in a T75 flask with complete DMEM (supplemented with 10% FBS and penicillin/streptomycin (100 U/ml)). BMDMs were cultured with a 30% L929 cell-conditioned medium in DMEM complete medium for 7 days before any further experimental procedure. L929 cells were purchased from ATCC (Manassas, VA). L929 cells were cultured in DMEM media with 10% FBS and 1% penicillin/streptomycin at 37°C in a 5% CO2 incubator.

### 2.4. Collection of Bronchoalveolar Lavage Fluids (BALF)

The mice were executed by exsanguination without any anesthetics, and the body was opened to expose the trachea. The trachea was infused by 1 ml PBS using a 20-g angiocatheter, and the PBS was collected for reperfusion. Repeat the process for 5 times, and the fluid was collected as BALF.

### 2.5. Isolation and Characterization of Exosomes

ADSCs (5 × 10^6^) were seeded in a 10 cm cell-culturing dish and cultured in DMEM supplied with 10% exosome-free FBS overnight. Next day, the ADSCs were transfected with miR-181-5p mimics (50 nmol/L) or cel-miR-67 mimics via 4 *μ*L Lipofectamine 2000 for 48 hours. Subsequently, the exosomes were isolated from the supernatant of ADSCs by using a Total Exosomes Isolation Kit (Invitrogen, USA) in accordance with the manufacturer's protocol. The exosomes were resuspended in PBS and stored in a -80°C refrigerator. The concentration of exosomes was measured by nanoparticle tracking analysis (NTA) on a NanoSight analyzer (Malvern, UK). Exosomes were identified by Western blotting detection of CD63 and CD9. For transmission electron microscopy (TEM), the exosomes were suspended in PBS and dropped on the cooper grids covered by carbon, washed, stained by uranyl acetate, and air dried. The images were taken by using a TEM (Philips, Netherland). Exosomes (2 *μ*g) suspended in 20 *μ*L PBS were used for cell treatment.

### 2.6. Western Blotting and ELISA

Cells or exosomes were homogenized by the RIPA lysis buffer containing a cocktail of protease inhibitors (Thermo). The total protein (30 *μ*g) was resolved in SDS-PAGE gels and shifted to the NC membranes, followed by incubation with primary antibodies and subsequent corresponding secondary antibodies. The bands were visualized by incubating with an enhanced chemiluminescence (ECL) reagent (Millipore, USA). ELISA kits were ordered from Thermo to detect the secretion of IL-1*β* and IL-18 in BALF under the manufacturer's instructions.

### 2.7. Real-Time PCR Assay

The levels of miR-181-5p in ADSCs, exosomes derived from ADSC, and exosome-treated BMDMs were detected by real-time PCR experiment. In brief, total RNA was isolated from exosomes by using the TRIzol solution, followed by reverse transcription to cDNAs by an Easy Script kit (TransGen, China). The relative expression of miR-181-5p was measured by using a SYBR Green SuperMix kit (TransGen) in ABI Prism 7900 (Applied Biosystems, USA) and normalized to the internal control U6. The primers were as follows: U6, sense, 5′-CTCGCTTGGGCAGCACA-3′, antisense, 5′-AACGCTTCACGAATTTGCGT-3′; miR-181-5p, sense, 5′-GCGCAACATTCAACGCTGTCG-3′, antisense, 5′-GTGCAGGGTCCGAGGT-3′.

### 2.8. Luciferase Reporter Gene Assay

We used the online tool TargetScan to predict the potential binding site between miR-181-5p and 3′UTR regions of STAT3. The 3′UTR regions of STAT3 containing wide type or mutated binding site with miR-181-5p were cloned into pGL3 vector to obtain the pGL3-STAT-WT (STAT3-WT) and pGL3-STAT-Mut (STAT3-Mut). 293T cells and BMDM were seeded in 12-well plates and were cotransfected with STAT3-WT or STAT3-Mut, miR-181-5p mimics or NC, and pRL-TK vector as internal control. After transfection for 24 hours, cells were lysed and subjected to a dual-luciferase reporter system (Promega, USA) to measure the luciferase activity according to the manufacturer's instruction.

### 2.9. Animal Model

C57BL/6 mice of both genders (6-week-old) were ordered from the Charles River Laboratories (USA) and divided randomly into control and experimental groups. To establish pneumonia model, mice were anesthetized and infected with LPS (1 *μ*g) or *K. pneu* (10^4^ CFU) through the airway. The freshly cultured or cryopreserved ADSCs were intravenously injected into mice after infection of *K. pneumoniae* for 6 hours. The mice were then sacrificed 16 hours or 48 hours after the inoculation of pneumonia. The blood and major organs (the lung, liver, and spleen) were collected to determine the levels of *K. pneu* and for following experiments. To determine the localization of exosomes in macrophages in the lung tissue, PKH26-labeled exosomes (70 *μ*g in 100 *μ*L PBS) were intratracheally given to the infected mice.

### 2.10. Immunofluorescence Staining and Histological Examination (H&E)

For confocal experiments, ADSCs were transfected with cyc3-labelled oligo to obtain exosomes. Then, the BMDM were treated with exosomes containing cyc3-labelled oligo and GW4869 (exosomal inhibitor). The cells were observed and photographed under a fluorescence microscope (Leica, Germany). To determine the localization of exosomes in the lung, the lung sections were incubated with anti-CD86 antibody (biomarker of macrophages) and anti-Ly-6G antibody (biomarker of neutrophiles), followed by incubation with Alexa 488 secondary antibody (Thermo) and DAPI. The images were photographed by the fluorescence microscope (Leica). Mouse lung tissues were also stained by haematoxylin and eosin (H&E) dye to observe tissue damage and captured by a microscope (Leica).

### 2.11. Inflammation and Caspase-1 Activity

To detect the inflammation occurred in the lung, we counted the number of inflammatory cells in BALF by cytospin. In short, the cell suspension was centrifuged for 5 minutes at a 300 rpm in a Cytospin machine (Thermo). The numbers of macrophages and neutrophils were counted by a hemocytometer. The inflammasome activity in the lung was detected by using a Caspase 1 Inflammasome Assay Kit (Thermo).

### 2.12. Ethical Guidelines

Animal care and method procedure were authorized by the Animal Ethics Committee of Wuxi Fifth People's Hospital (Approval No. 2020-0317-16). The procedures conformed to the Guide for the Care and Use of Laboratory Animal published by the US National Institutes of Health (NIH publication, 8th edition, 2011).

### 2.13. Statistical Analysis

Data in this work were analyzed by the SPSS 22.0 software. The differences between two or more groups were analyzed by two-tailed unpaired Student's *t*-test or the one-way ANOVA analysis. Parametric data are shown as means with standard deviation (SD).

## 3. Results

### 3.1. Identification of Human Adipose-Derived Mesenchymal Stem Cells (ADSCs)

**Figure 1:**
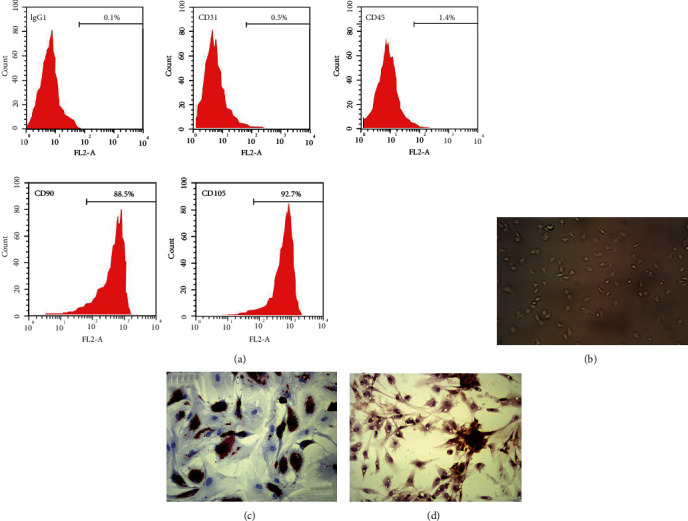
Identification of human adipose-derived mesenchymal stem cells (ADSCs). (a) The surface markers of ADSCs were detected by flow cytometry analysis. (b) The morphology of ADSC was shown. (c) The ADSCs were cultured in adipogenesis differentiation medium for 14 days and were analyzed by Oil Red O staining. (d) The ADSCs were cultured in osteogenesis differentiation medium for 21 days and were analyzed by Alizarin Red S staining. *N* = 3.

We first successfully isolated and characterized the ADSCs from adipose tissue of mice. Flow cytometry for examination of cell surface biomarkers demonstrated that ADSCs expressed CD90 and CD105, the typical biomarkers of ADSCs, rather than CD31 and CD45 (Figure 1(a)). The morphology of ADSC was captured by a microscope and was shown in Figure 1(b). The notable differentiation of ADSCs towards adipocytes and osteoblasts was observed by Oil Red O staining (Figure 1(c)) and Alizarin Red S staining (Figure 1(d)), respectively.

### 3.2. Exosomal miR-181a-5p Communication between Adipose-Derived Mesenchymal Stem Cells (ADSCs) and BMDM

**Figure 2:**
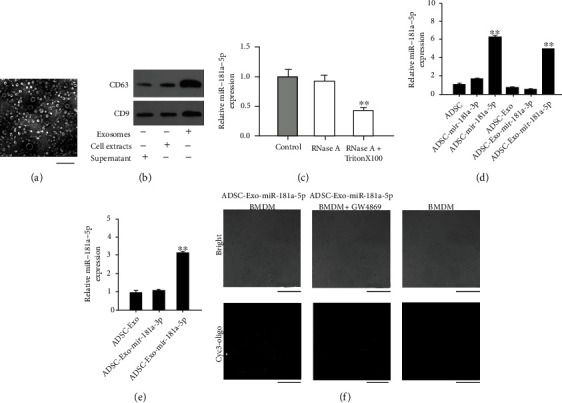
Exosomal miR-181a-5p communication between adipose-derived mesenchymal stem cells (ADSCs) and BMDM. (a) The exosomes from ADSCs were observed by TEM. Scale bar = 50 *μ*m. (b) The expression of CD63 and CD9 was detected by Western blot analysis. (c) The BMDM was treated with exosomes from ADSCs and treated with RNaseA with or without Triton X100. The expression of miR-181a-5p was measured by qPCR. (d) The ADSCs were treated with miR-181a-5p or miR-181a-3p mimic. The expression of miR-181a-3p was analyzed by qPCR in ADSCs or exosomes from ADSCs. (e) The ADSCs were treated with miR-181a-5p or miR-181a-3p and the BMDM was treated with exosomes from the ADSCs. The expression of miR-181a-5p was assessed by qPCR in BMDM. (f) The ADSCs were treated with miR-181a-5p and the BMDM was treated with exosomes from the ADSCs or cotreated with GW4869 (10 M for 48 hours). The miR-181a-5p stained with cyc3-oligo in BMDM was observed by Brightfield and immunofluorescence using confocal analysis. Scale bar = 75 *μ*m. *N* = 3. Data are presented as mean ± SD. Statistic significant differences were indicated: ^∗∗^*P* < 0.01.

We then explored the transferring of miR-18a-5p from ADSCs to BMDM. The exosomes isolated from ADSCs were identified by TEM, and the exosome protein markers CD63 and CD9 were validated by Western blot analysis (Figures 2(a) and 2(b)). Next, the BMDM was treated with exosomes from ADSCs and RNaseA with or without the administration of Triton X100. The expression of miR-18a-5p was reduced in the BMDM cotreated with RNaseA and Triton X100, comparing with BMDMs treated by RNaseA alone (Figure 2(c)). Moreover, the ADSCs were treated with miR-181a-5p or miR-181a-3p mimic and the expression of miR-181a-5p was elevated by miR-181a-5p mimic but not miR-181a-3p mimic, both in the ADSCs and exosomes from ADSCs (Figure 2(d)). The exosomes isolated from ADSCs after transfection with miR-181a-5p or miR-181a-3p were administrated to BMDM. Notably, the level of miR-181a-5p was also enhanced in BMDM treated with exosomes from miR-181a-5p mimic-treated ADSCs (Figure 2(e)). Besides, the transfer and uptake of miR-181a-5p from miR-181a-5p mimic-treated ADSCs to BMDM were visualized in BMDM by cyc3 staining, and the exosome inhibitor GW4869 could inhibit the uptake (Figure 2(f)). These results demonstrated the potential communication between ADSCs and BMDM via exosomes.

### 3.3. *Klebsiella* Pneumonia Infection Enhances miR-181a-5p Expression in BALF and BMDM

**Figure 3:**
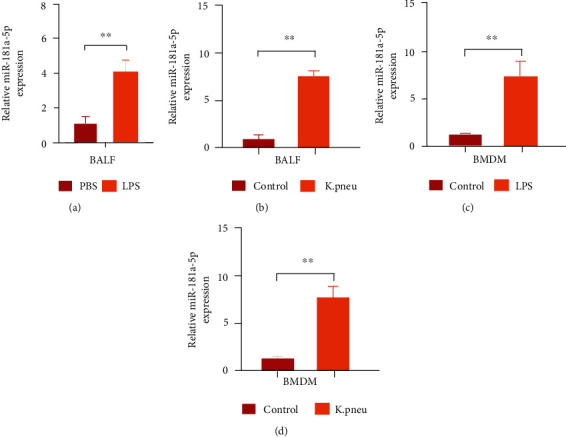
*Klebsiella* pneumonia infection enhances miR-181a-5p expression in BALF and BMDM. (a) The mice were treated with PBS or LPS (1 *μ*g). The expression of miR-181a-5p was measured by qPCR in BALF. (b) The mice received PBS or *Klebsiella pneumoniae* (10^4^ CFUs). The expression of miR-181a-5p was detected by qPCR in BALF. (c) The BMDM was treated with LPS (100 ng/mL) and the expression of miR-181a-5p was assessed by qPCR in BMDM. (d) The BMDM was incubated with *K. pneumoniae* treatment (10^7^ CFU bacteria/10^5^ macrophages) and the expression of miR-181a-5p was determined by qPCR in BMDM. *N* = 5. Data are presented as mean ± SD. Statistic significant differences were indicated: ^∗∗^*P* < 0.01.

We further determined the alteration of miR-181a-5p upon LPS-induced inflammatory response. As was indicated by real-time PCR assay, the expression of miR-181a-5p was elevated in the bronchoalveolar lavage fluid (BALF) from the mice treated with LPS (Figure 3(a)). Consistently, the *K. pneu* infection induced the miR-181a-5p expression in BALF from the mice (Figure 3(b)). Meanwhile, the expression of miR-181a-5p was increased in BMDM upon LPS treatment (Figure 3(c)). Similarly, the *K. pneu* infection stimulated the expression of miR-181a-5p in BMDM (Figure 3(d)).

### 3.4. MiR-181a-5p Is Able to Target STAT3 Signaling

**Figure 4:**
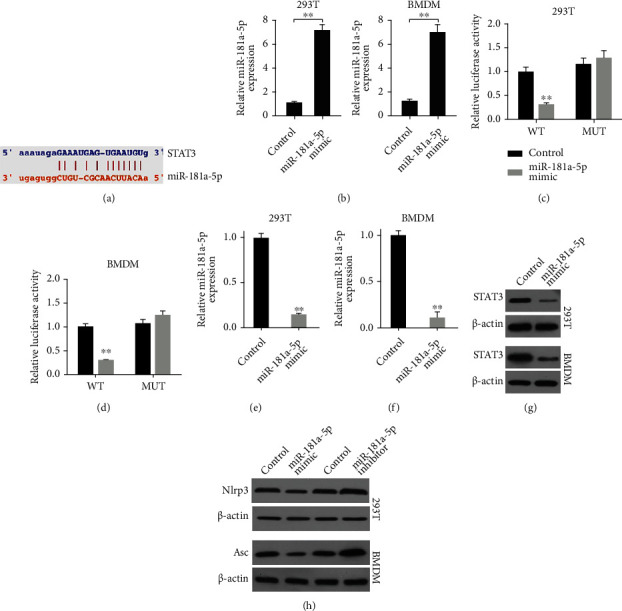
MiR-181a-5p is able to target STAT3 signaling. (a) The binding of miR-181a-5p and STAT3 mRNA 3′ UTR was predicted in ENCORI website. (b–g) The 293T cells and BMDM were treated with control mimic or miR-181a-5p mimic. (b) The expression of miR-181a-5p was detected by qPCR. (c and d) The luciferase activities of STAT3 mRNA 3′ UTR were measured by luciferase reporter gene assays. (e and f) The mRNA expression of STAT3 was analyzed by qPCR assays. (g) The protein expression of STAT3 was tested by Western blot analysis. (h) The protein expression of Nlrp3 and Asc was examined by Western blot analysis. *N* = 3. Data are presented as mean ± SD. Statistic significant differences were indicated: ^∗∗^*P* < 0.01.

Next, we identified a potential interaction of miR-181a-5p and STAT3 mRNA 3′ UTR in a bioinformatic analysis (Figure 4(a)). The treatment of miR-181a-5p mimic significantly enhanced miR-181a-5p expression in both 293T cells and BMDM (Figure 4(b)). Meanwhile, the luciferase activities of STAT3 mRNA 3′ UTR were repressed by miR-181a-5p mimic in 293T cells and BMDM (Figures 4(c) and 4(d)). Consistently, the mRNA and protein expression of STAT3 were reduced by miR-181a-5p mimic in 293T cells and BMDM (Figures 4(e)–4(g)). Moreover, the treatment of miR-181a-5p mimic inhibited while miR-181a-5p mimic inhibitor enhanced expression of Nlrp3 and Asc, the key components of Nlrp3 inflammasome, in 293T cells and BMDM (Figure 4(h)). These data indicated the anti-inflammatory effect of miR-181a-5p via regulating STAT3 signaling.

### 3.5. ADSC-Derived Exosomal miR-181a-5p Represses Pulmonary Outgrowth and Dissemination of *Klebsiella* Pneumonia Infection

**Figure 5:**
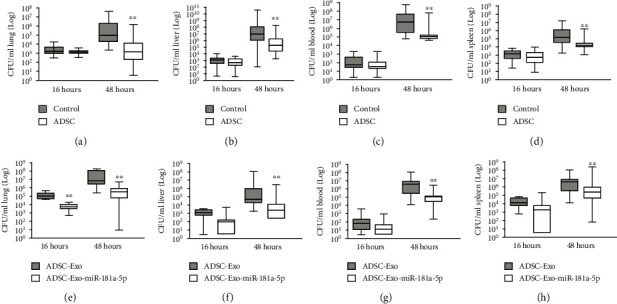
ADSC-derived exosomal miR-181a-5p represses pulmonary outgrowth and dissemination of *Klebsiella* pneumonia infection. (a–d) The mice received PBS or *Klebsiella pneumoniae* (10^4^ CFUs). And the mice were treated with ADSCs. The CFU loads in the lung (a), liver (b), blood (c), and spleen (d) were analyzed. (e–h) The mice received PBS or *Klebsiella pneumoniae* (10^4^ CFUs). And the mice were treated with exosomes from ADSCs or exosomes from miR-181a-5p-treated ADSCs. The CFU loads in the lung (a), liver (b), blood (c), and spleen (d) were analyzed. *N* = 5. Data are presented as mean ± SD. Statistic significant differences were indicated: ^∗∗^*P* < 0.01.

Subsequently, we evaluated the effect of ADSC-derived exosomal miR-181a-5p on *K. pneu* infection-induced lung inflammation in vivo. The mice received *K. pneu* infection followed by treatment with ADSCs, exosomes from ADSCs or exosomes from miR-181a-5p-treated ADSCs. Our data showed that the treatment of ADSCs reduced the CFU loads of *K. pneu* infection in the lung, liver, blood, and spleen (Figures 5(a)–5(d)). Meanwhile, the treatment of exosomes from miR-181a-5p-treated ADSCs attenuated the CFU loads of *K. pneu* infection in the lung, liver, blood, and spleen compared with exosomes from ADSCs (Figures 5(e)–5(h)).

### 3.6. ADSC-Derived Exosomal miR-181a-5p Attenuates the *Klebsiella* Pneumonia Infection-Induced Lung Inflammation in a Macrophage-Related Manner

**Figure 6:**
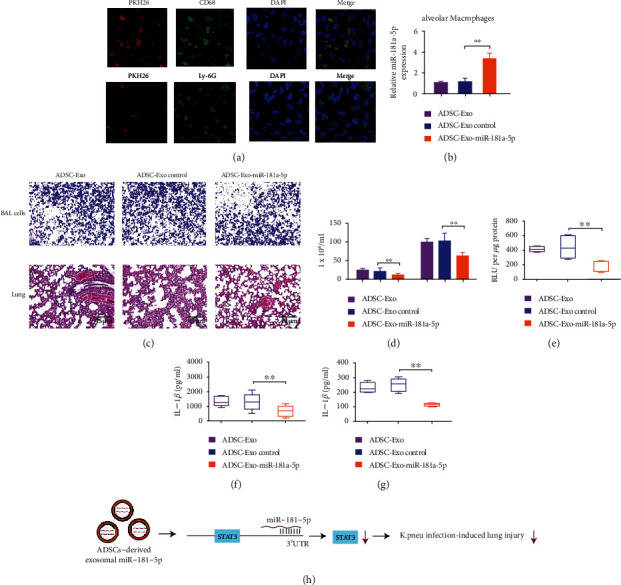
ADSC-derived exosomal miR-181a-5p attenuates the *Klebsiella* pneumonia infection-induced lung inflammation. (a–g) The mice received PBS or *Klebsiella pneumoniae* (10^4^ CFUs). And the mice were treated with exosomes from ADSCs or exosomes from miR-181a-5p-treated ADSCs. (a) The PKH26-labelled miR-181a-5p, CD68, and Ly-6G were detected by immunofluorescence staining. (b) The expression of miR-181a-5p was measured by qPCR in macrophages from BALF. (c) BALF cells and lung sections were analyzed by H&E staining. (d) The numbers of macrophages or neutrophils from BALF were counted. (e) The activity of caspase-1 in murine lung tissue was determined. (f and g) The levels of IL-1*β* and IL-18 were tested by ELISA in BALF. *N* = 5. Data are presented as mean ± SD. Statistic significant differences were indicated: ∗∗*P* < 0.01. (h) ADSC-derived exosomal miR-181a-5p alleviated *K. pneu* infection-induced lung injury by targeting STAT3 signaling.

We next investigated inflammatory response during exosomal miR-181a-5p regulated anti-*K. pneu* process. We observed that the majority of PKH26-positive cells were CD68 positive (monocyte lineage marker) but not the Ly-6G/Ly-6C (polymorphonuclear neutrophils marker), indicating that exosomes were specifically taken by lung macrophages (Figure 6(a)). The treatment of miR-181a-5p enhanced the miR-181a-5p expression in macrophages obtained from BALF (Figure 6(b)). ADSC-derived exosomal miR-181a-5p repressed cellular infiltration in the lung tissue (Figure 6(c)). The cell counts of macrophages and neutrophils were decreased by ADSC-derived exosomal miR-181a-5p ((Figure 6(d))). ADSC-derived exosomal miR-181a-5p attenuated the inflammasome activity and the levels of IL-1*β*, IL-18, TNF-*α*, TGF-*β*, and IL-8 in the lung (Figures 6(e)–6(g) and Figure S1).

## 4. Discussion

*Klebsiella pneumoniae* is a leading cause of gram-negative pneumonia. ADSCs have demonstrated inhibitory function in *K. pneu* infection-induced injury. Nevertheless, the effect of miR-181a-5p from ADSC-derived exosomes on *Klebsiella pneumoniae* infection-induced lung injury is still obscure. In the present study, we identified the critical role of ADSC-derived exosomal miR-181a-5p in attenuating *Klebsiella* pneumonia infection-induced lung injury.

Previous study showed that miR-181a-5p relieves inflammation by targeting endocan in monocrotaline-stimulated pulmonary arterial hypertension [29]. MiR-181a-5p regulates acute lung injury and respiratory distress syndrome [30]. MiR-181a-5p restricts vascular atherosclerosis and inflammation [31]. Here, in this research, our data showed that the miR-181a-5p expression was elevated by LPS or *K. pneu* infection in BMDM and the bronchoalveolar lavage fluid (BALF) from the mice. These data implied that miR-181a-5p may play a critical role in modulation of *K. pneu* infection. Meanwhile, microvesicle- (MV-) secreted miR-223/142 is enhanced in BALF and inhibits lung inflammation and macrophage activation by inactivating Nlrp3 inflammasome in *K. pneu* infection [22]. MiR-23a and MiR-155 regulate *Klebsiella pneumoniae* adhesion to human pulmonary epithelial cells by targeting integrin *α*5*β*1 signaling [23]. Perlee and colleagues recently reported that ADSCs regulate lung immunity to enhance antibacterial response in *K. pneu* infection-caused pneumosepsis [14]. In this study, we observed that the transfer and uptake of miR-181a-5p from miR-181a-5p mimic-treated ADSCs to BMDM and the exosome inhibitor GW4869 could inhibit the uptake. ADSC-derived exosomal miR-181a-5p repressed pulmonary outgrowth and dissemination of *K. pneu* infection in mice, as well as attenuated the *K. pneu* infection-induced lung inflammation in vivo. Our data provide a critical mechanism of ADSCs preventing *K. pneu* infection-induced lung injury and indicate a new function of miR-181a-5p. The specific role of miR-181a-5p in BMDM during *K. pneu* infection is still unclear and requires further investigation in the future. Moreover, STAT3 has been revealed to play important roles in *K. pneu* infection-related injury [32, 33]. Here, we identified that miR-181a-5p was able to inhibit STAT3 expression at posttranscriptional levels and repressed Nlrp3 and Asc expression in BMDM. Our finding provides new insight into the mechanism *K. pneu* infection-induced lung injury and presents the unreported correlation of miR-181a-5p with STAT3 signaling during this process. Meanwhile, there are some limitations of the current study. The success of exosome isolation should be validated by size-based method, such as dynamic light scattering (DLS). The clinical value of ADSC-derived exosomal miR-181a-5p was not evaluated in this investigation, and the delivery approaches and effectiveness of ADSC-derived exosomal miR-181a-5p in the clinical application should be explored due to the unstable properties of miRNAs. In addition, STAT3 may be just one of the downstream factors of miR-181a-5p in the modulation of *K. pneu* infection-induced lung injury and other potential mechanisms need to be investigated in future studies.

## 5. Conclusions

In conclusion, we concluded that ADSC-derived exosomal miR-181a-5p alleviated *K. pneu* infection-induced lung injury by targeting STAT3 signaling (Figure 6(h)). ADSC-derived exosomal miR-181a-5p may serve as a potential candidate for the treatment of *Klebsiella* pneumonia infection-induced lung injury.

## Data Availability

The data that support the findings of this study are available from the corresponding author upon reasonable request.

## Supplementary Materials

Supplementary Materials Figure S1: ADSC-derived exosomal miR-181a-5p attenuates the *Klebsiella* pneumonia infection-induced lung inflammation. (A–C) The mice received PBS or *Klebsiella pneumoniae* (10^4^ CFUs). And the mice were treated with exosomes from ADSCs or exosomes from miR-181a-5p-treated ADSCs. The levels of TNF-*α*, TGF-*β*, and IL-8 were tested by ELISA in BALF. *N* = 5. Data are presented as mean ± SD. Statistic significant differences were indicated: ^∗∗^*P* < 0.01.
