# Supplementary figures and images for: MiR-181a-5p Delivered by Adipose-Derived Mesenchymal Stem Cell Exosomes Alleviates Klebsiella pneumonia Infection-Induced Lung Injury by Targeting STAT3 Signaling
Source: Mediators Inflamm. 2022 Dec 14;2022:5188895. doi: 10.1155/2022/5188895 (PMC9771653; doi:10.1155/2022/5188895)

Figure S1

**A**

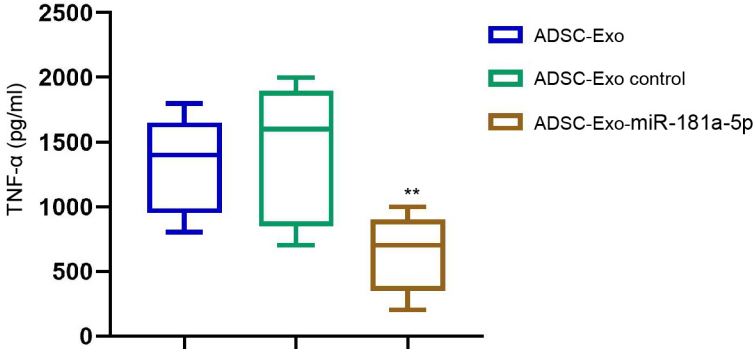

**B**

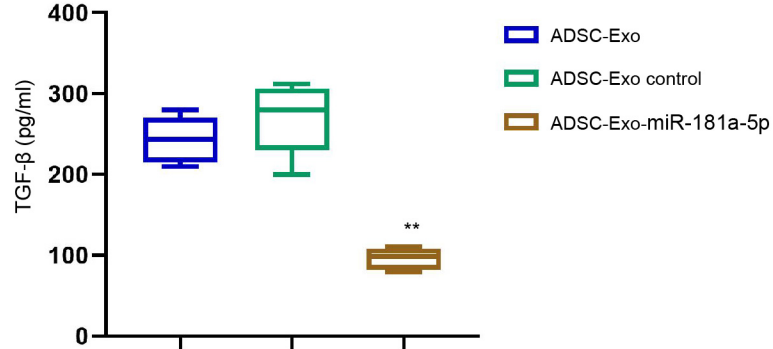

**C**

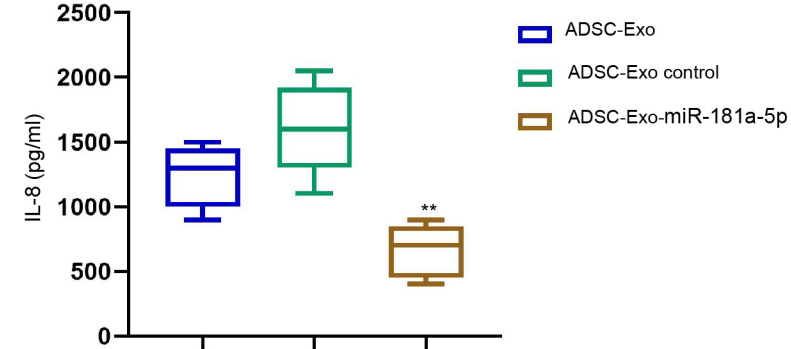

Supplement: Supplementary Materials — Figure S1: ADSC-derived exosomal miR-181a-5p attenuates the Klebsiella pneumonia infection-induced lung inflammation. (A–C) The mice received PBS or Klebsiella pneumoniae (104 CFUs). And the mice were treated with exosomes from ADSCs or exosomes from miR-181a-5p-treated ADSCs. The levels of TNF-α, TGF-β, and IL-8 were tested by ELISA in BALF. N = 5. Data are presented as mean ± SD. Statistic significant differences were indicated: ∗∗P < 0.01. [file 5188895.f1.pdf]
